# TccP4: a novel effector identified in the *Escherichia albertii* strain 1551-2 required for attaching and effacing lesion formation on infected Nck-null cells

**DOI:** 10.1128/spectrum.02055-24

**Published:** 2025-01-29

**Authors:** Iranildo do A. Fernandes, Tadasuke Ooka, Daiany R. P. de Lira, Fernando H. Martins, Henrique Orsi, Nina Jones, Waldir P. Elias, Tetsuya Hayashi, Tânia A. T. Gomes, Rodrigo T. Hernandes

**Affiliations:** 1 Instituto de Biociências, Universidade Estadual Paulista (UNESP), Botucatu, Brazil; 2 Department of Microbiology, Graduate School of Medical and Dental Sciences, Kagoshima University, Kagoshima, Japan; 3 Department of Biochemistry, University of Texas Southwestern Medical Center, Dallas, Texas, USA; 4 Department of Microbiology, University of Texas Southwestern Medical Center, Dallas, Texas, USA; 5 Department of Molecular and Cellular Biology, University of Guelph, Guelph, Ontario, Canada; 6 Laboratório de Bacteriologia, Instituto Butantan, São Paulo, Brazil; 7 Department of Bacteriology, Faculty of Medical Sciences, Kyushu University, Fukuoka, Japan; 8 Departamento de Microbiologia, Imunologia e Parasitologia, Escola Paulista de Medicina, Universidade Federal de São Paulo (EPM - UNIFESP), São Paulo, Brazil; Cinvestav-IPN, Mexico City, Mexico

**Keywords:** TccP4, AE lesion, T3SS and diarrhea

## Abstract

*Escherichia albertii* is a pathogen that causes sporadic cases and outbreaks of diarrhea. The main virulence feature of this bacterium is the attaching and effacing (AE) lesion formation on infected intestinal epithelial cells, which is characterized by the formation of pedestal-like structures that are rich in F-actin. The Brazilian *E. albertii* 1551-2 strain can recruit F-actin using both the Nck-dependent and the Nck-independent pathways, the latter of which uses an adaptor protein named Tir-cytoskeleton coupling protein (TccP/EspF_U_). Genome analyses of the 1551-2 strain unveiled the existence of a gene encoding a putative novel TccP subtype in addition to a gene encoding for the TccP3 subtype. Amino-acid sequence comparison with known TccP subtypes (TccP/EspF_U_, TccP2, and TccP3) confirmed that the protein represents a novel TccP subtype—named here TccP4. Lack of TccP4 led to an approximately 96% reduction in the ability of the *tccP3* deletion mutant of strain 1551-2 to induce the F-actin-rich pedestals formation in the infected Nck-null mouse embryonic fibroblasts (MEF) cells. The *tccP4* gene was distributed widely in *E. albertii*, including the strains first separated from other *E. albertii* strains, suggesting that this gene was acquired at a very early stage during the diversification of *E. albertii*. The highly variable genetic organization of the *tccP4*-containing regions and the presence of various mobile genetic elements in this region may explain the lack of *tccP4* in *E. albertii* strains belonging to various lineages.

**IMPORTANCE:**

*E. albertii*, one of the new members of the genus *Escherichia,* is a diarrheagenic pathogen. The main characteristic of its pathogenicity is the formation of attaching and effacing (AE) lesions on the surface of infected epithelial cells. Here we identified a novel subtype of the TccP type 3 secretion system (T3SS) effector family (termed TccP4), which is required for the recruitment of F-actin during the AE lesion formation in infected host cells by the *E. albertii* 1551-2 strain. We also revealed that TccP4 is unique to *E. albertii* and widely distributed in this species, suggesting that the *tccP4* gene was acquired at a very early stage during the diversification process of *E. albertii*. These findings expand our understanding of the function and diversity of this important T3SS effector family.

## INTRODUCTION

The species *Escherichia albertii* represents one of the new members of the genus *Escherichia* that is responsible for sporadic cases, as well as outbreaks of diarrhea, in several countries (1–4). *E. albertii* colonizes the human intestinal mucosa and, similarly to enteropathogenic (EPEC) and enterohemorrhagic (EHEC) *E. coli,* induces the attaching and effacing (AE) lesion formation in the host cells (5–8). The AE lesion is characterized by intimate adherence, microvilli destruction, and the formation of a pedestal-like structure that is rich in F-actin and other eukaryotic cytoskeleton elements (9, 10).

The genes encoding proteins involved in the AE lesion formation are located within a pathogenicity island (PAI) termed the locus of enterocyte effacement or LEE region (11). The LEE region is organized in five polycistronic operons, termed LEE1 to LEE5, two bicistronic operons (*espG-rorf1* and *grlA-grlR*), and four individual genes (*etgA*, *cesF*, *map,* and *escD*) (12). This PAI encodes a type 3 secretion system (T3SS), as well as the adhesin intimin (13) and Tir (translocated intimin receptor) which is the intimin receptor translocated to host cells via the T3SS (14).

During the establishment of the AE lesion, the recruitment of F-actin for pedestal formation in host cells can occur via two distinct pathways in which the participation of the eukaryotic adapter protein Nck (non-catalytic region of tyrosine kinase) may be necessary or not (15). These two pathways have been referred to in the literature as Nck-dependent and Nck-independent (13, 16, 17). In the Nck-dependent pathway, first described in the typical EPEC prototype strain E2348/69 (14, 18), the tyrosine residue 474 (Y_474_) present in the C-terminal domain of the Tir protein is phosphorylated by host cell kinases triggering the recruitment of Nck to the bacterial adherence site. Subsequently, the actin nucleation-promoting factor neuronal Wiskott-Aldrich syndrome protein (N-WASP) and the actin-related protein 2/3 (Arp2/3) complex are sequentially activated, initiating the actin polymerization processes (18, 19).

On the other hand, the Tir protein produced by EHEC strains of serotype O157:H7 lacks the Y_474_ residue and, therefore, this pathogen does not use the Nck adaptor protein to recruit actin for the AE lesion formation (16). Instead, in an Nck-independent pathway, EHEC O157:H7 requires another bacterial effector translocated by the T3SS, termed Tir-cytoskeleton coupling protein (TccP) or EspF_U_, which is linked to the C-terminal domain of Tir via the eukaryotic adaptor protein insulin receptor tyrosine kinase substrate (IRTKS), resulting in the activation of N-WASP and Arp2/3 (17, 20, 21). Of note, IRTKS and insulin receptor substrate of 53 kDa protein (IRSp53) are related proteins serving as adaptors of F-actin recruitment (22). So far, three distinct subtypes of TccP/EspF_U_ have been described and designated as follows: TccP/EspF_U_ (referred to as TccP1 in this study), TccP2, and TccP3 (17, 20, 23, 24).

Previous studies have shown that the Brazilian *E. albertii* prototype 1551-2 strain can employ both the Nck-dependent (25) and the Nck-independent pathways for F-actin polymerization during AE lesion formation (24). Even though TccP3 was first described in the 1551-2 strain, a *tccP3* deletion mutant preserved its ability to recruit F-actin in an Nck-independent pathway in assays performed with mouse embryonic fibroblasts lacking Nck (Nck-null MEF cells) (24).

Further, genomic analyses searching for genes with the potential to encode novel T3SS effectors revealed that the *E. albertii* 1551-2 strain harbors a gene encoding a novel TccP subtype termed, in the present study, *tccP4*. In view of this scenario, our main objective was to evaluate the contribution of TccP4 in the AE lesion formation in an Nck-independent pathway, as well as understand the distribution of the *tccP4* gene among other sequenced *E. albertii* strains.

## RESULTS

### TccP4: a novel TccP subtype identified in the *E. albertii* 1551-2 strain

**Fig 1:**
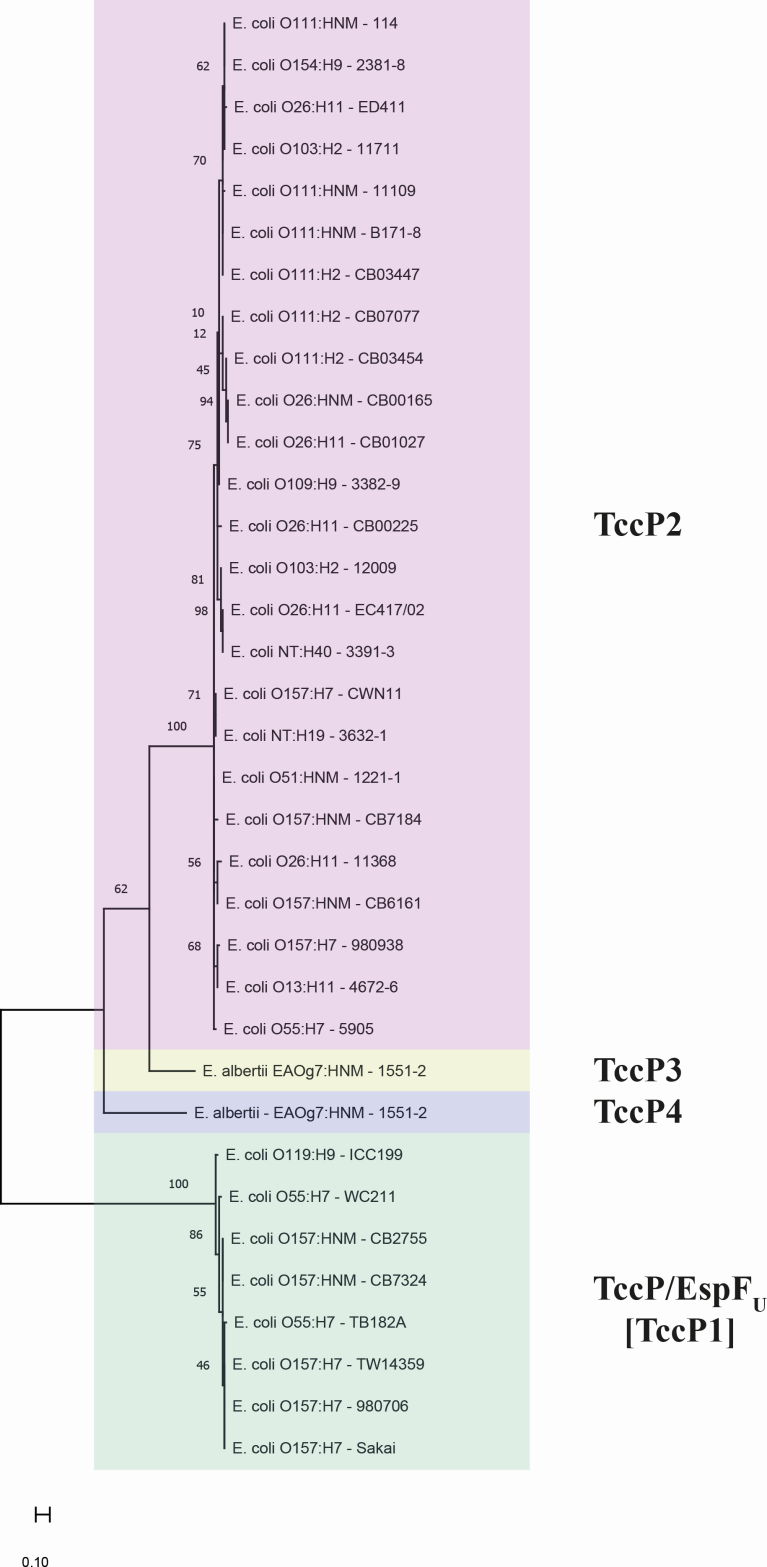
Identification of a novel TccP subtype—termed TccP4—in the *E. albertii* 1551-2 strain. A phylogenetic analysis of the amino acid sequences of the distinct TccP subtypes (TccP1, TccP2, and TccP3), using the maximum likelihood method, showed four main branches representing the three TccP subtypes described to date, as well as the novel TccP subtype—TccP4—identified in this study. Each TccP subtype is indicated by a specific color as follows: green: TccP/EspF (TccP1), red: TccP2, yellow: TccP3, and blue: the novel TccP subtype—TccP4.

During the genomic analysis of the *E. albertii* 1551-2 strain, we identified a gene able to potentially encode a novel TccP subtype with 227 amino acids in length and approximately 24 kDa in molecular weight (Table S1; supplemental material is found at DOI 10.6084/m9.figshare.28152620). To test this hypothesis, its amino acid sequence (Protein ID: AUS64398.1) was employed to carry out a phylogenetic analysis through the construction of a maximum likelihood (ML) tree, as well as to determine the percentage of amino acid sequence identity between different subtypes of TccP described to date (TccP1, TccP2, and TccP3). In the phylogenetic tree constructed, TccP proteins were separated into four main branches, three of which were represented by the three TccP subtypes already described in the literature (TccP1, TccP2, and TccP3), while the fourth branch represented the novel TccP subtype—termed TccP4—identified in this study (Fig. 1). Furthermore, TccP4 showed 60% or less of identity to the representative amino acid sequences of other TccP subtypes (Table S1; supplemental material is found at DOI 10.6084/m9.figshare.28152620), thus providing additional evidence that the protein is a novel TccP subtype.

**Fig 2:**
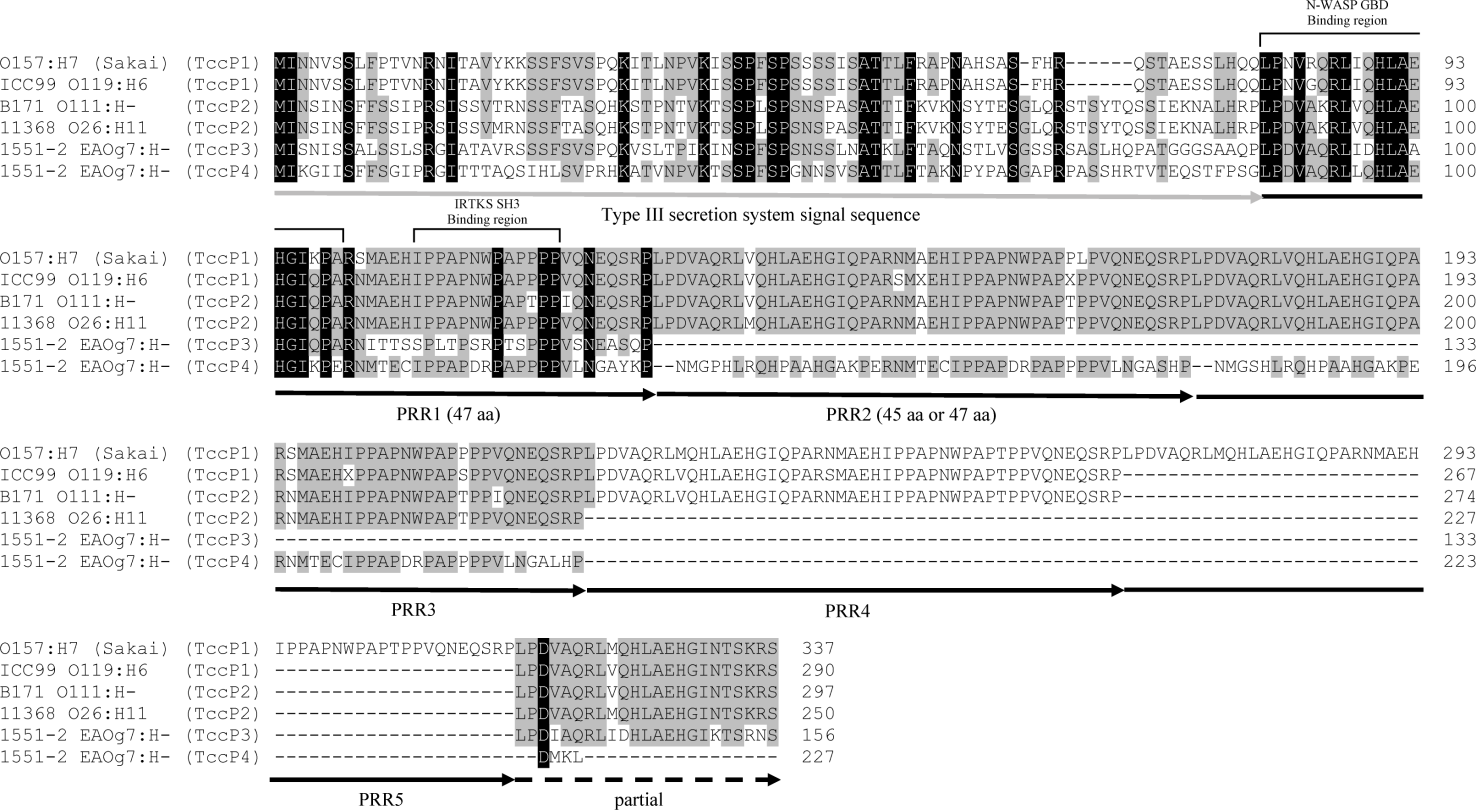
Amino acid sequence comparison of the TccP4 protein, identified in the *E. albertii* 1551-2 strain, with previously described TccP subtypes. The gray arrow indicates the type III secretion system signal sequence of the TccP family proteins. Proline-rich repeats (PRRs) are indicated by black arrows, while a partial PRR is indicated by a dashed black arrow. Amino acids marked in black and gray indicate amino acid residues, perfectly and partially conserved among the TccP subtypes, respectively. The type III secretion system signal sequence and the PRRs were marked as previously identified in the TccP/EspF_U_ (also referred to as TccP1) protein from O157:H7 enterohemorrhagic *E. coli* (26).

TccP4 of the 1551-2 strain harbored three proline-rich repeats (PRRs). While the first one was 47 amino acids in length and showed a high sequence identity to the PRRs of other TccP subtypes, the second and third ones were 45 amino acids in length and their sequences were divergent from the first PRR, as well as from the PRR present in other TccP subtypes (Fig. 2). In addition, TccP4 of 1551-2 strain lacked the partial PRR located at the C-terminus observed in the other subtypes.

### TccP4 is required for attaching and effacing lesion formation in infected host cells

To evaluate the functionality of TccP4 in the recruitment of F-actin for AE lesion formation, we generated a *tccP4* deletion mutant in the 1551-2Δ*tccP3* background, thus generating the 1551-2Δ*tccP3/tccP4* double mutant. The replacement of *tccP4* by the zeocin-encoding resistance gene (*ble*) was confirmed by Sanger sequencing (data not shown). The 1551-2Δ*tccP3/tccP4* double mutant carrying pTccP4 was also generated (Table 1), in which the expression of the recombinant TccP4-Myc protein was confirmed by immunoblotting using monoclonal α-Myc antibodies (Fig. S1; supplemental material is found at DOI 10.6084/m9.figshare.28152620). All four strains used in the subsequent steps of this study exhibited similar growth rates, as evidenced by the bacterial growth curves (Fig. S2A; supplemental material is found at DOI 10.6084/m9.figshare.28152620).

**TABLE 1:** Bacterial strains and plasmids used in this study

Strains and plasmids	Relevant characteristics	Reference
Strains		
1551-2	*E. albertii* (EAOg7:HNM)^*a*^ isolated from a child with diarrhea in Brazil (Nal^R^)	(27, 28)
1551-2*ΔtccP3*	1551-2 *tccP3* mutant (Nal^R^, Clo^R^)	(24)
1551-2*ΔtccP3*/*ΔtccP4*	1551-2 *tccP3*/*tccP4* double mutant (Nal^R^, Clo^R^, Zeo^R^)	This study
1551-2*ΔtccP3*/*ΔtccP4* (pTccP4)	1551-2*ΔtccP3*/*ΔtccP4* double mutant *in trans* complemented with the pTccP4 plasmid (Nal^R^, Clo^R^, Zeo^R^, Km^r^)	This study
1711-4Δ*fliC*	Source of zeocin cassette	(29)
Plasmids		
pKOBEG-Apra	Red recombinase system plasmid, Apra^R^	(30)
pKC471	pK187 vector harboring the *tccP/espF_U_* gene from the EHEC O157:H7 EDL933 cloned upstream the gene encoding the Myc epitope (Km^R^)	(17)
pTccP4	pKC471 vector harboring the *tccP4* gene, from 1551-2, replacing the *tccP/espF_U_* gene (Km^R^)	This study

^*a*^ EAOg: *Escherichia albertii* O-genotype; HNM: non-motile.

Nck-null MEF cells were used to evaluate the role of the TccP4 protein in F-actin recruitment for AE lesion formation in a Nck-independent pathway. First, we tested two prototype *E. coli* strains, E2348/69 (that uses the Nck-dependent pathway) and EDL933 (that uses the Nck-independent pathway), in both HeLa and Nck-null MEF cells. As expected, while both strains induced the recruitment of F-actin in HeLa cells, only the EDL933 strain, which produces the adapter protein TccP/EspF_U_ (referred to in the present study as TccP1), was able to trigger F-actin to the bacterial adherence site in Nck-null MEF cells (Fig. S3; supplemental material is found at DOI 10.6084/m9.figshare.28152620).

**Fig 3:**
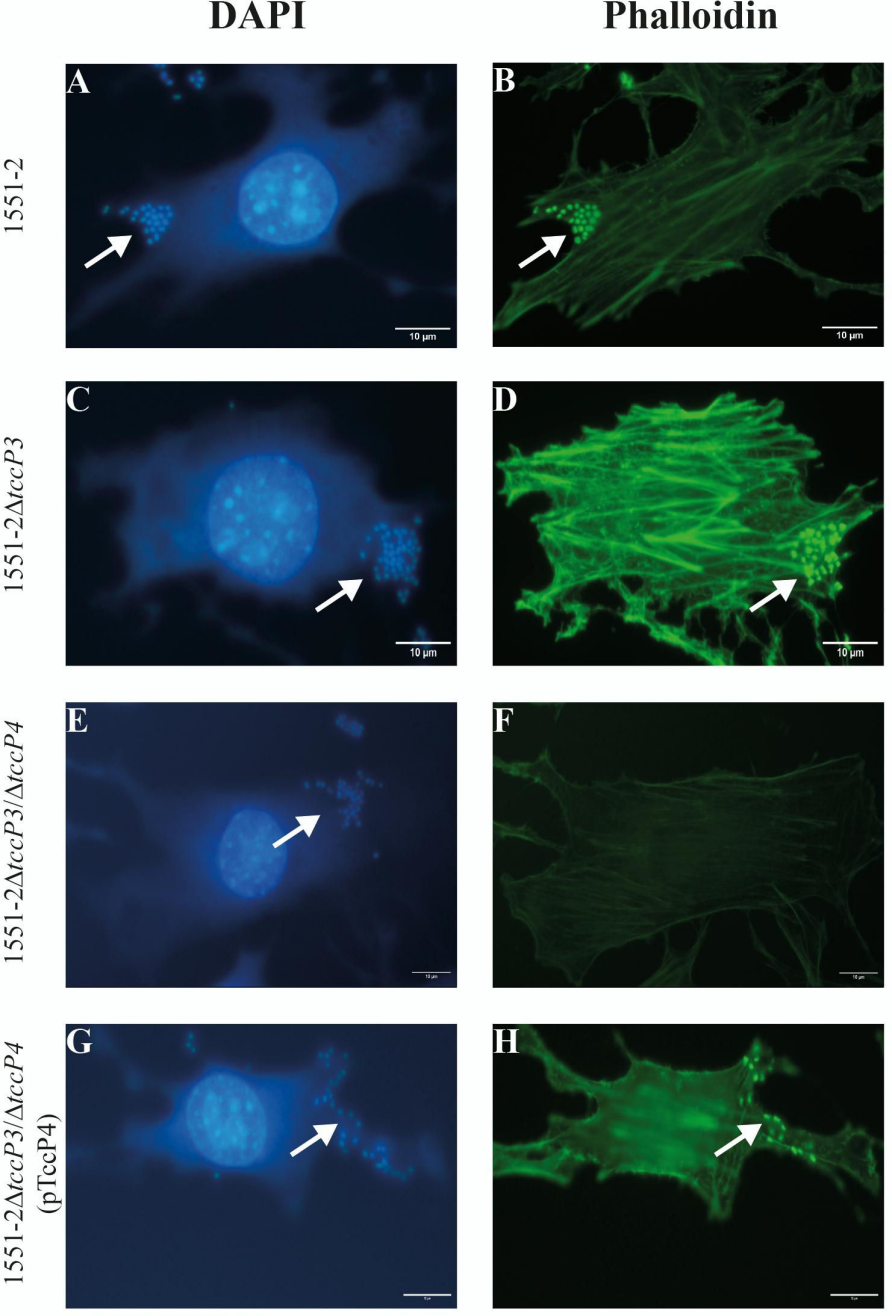
TccP4 is required for F-actin polymerization by 1551-2 strain in Nck-null MEF cells. Host cells and adherent bacteria were visualized with DAPI (4′,6-diamidino-2-phenylindole dihydrochloride) staining (A, C, E, and G), while the phalloidin staining allowed the visualization of polymerized F-actin underneath adherent bacteria (**B, D, and H**). Of importance, the *E. albertii* 1551-2Δ*tccP3*/Δ*tccP4* double mutant was unable to trigger F-actin for AE lesion formation in infected Nck-null MEF cells (**F**), thus indicating the importance of the TccP4 protein for the establishment of this phenotype. White arrows in panels A, C, E, and G indicate bacteria adhered to the host cells, while in panels B, D, and H indicate polymerized F-actin. Scale bar: 10 µm.

First, we confirmed that both 1551-2 and the 1551-2Δ*tccP3* strains were able to produce F-actin polymerization underneath the adherent bacteria in infected Nck-null MEF cells (Fig. 3). By contrast, we observed that the 1551-2Δ*tccP3/tccP4* double mutant was unable to polymerize F-actin for AE lesion formation in Nck-null MEF cells. This phenotype was restored in the complemented 1551-2Δ*tccP3/tccP4*(pTccP4) strain (Fig. 3). As a control, we observed that the additional deletion of the *tccP4* gene in the 1551-2Δ*tccP3* mutant did not affect its ability to induce F-actin polymerization in HeLa cells (Fig. S4; supplemental material is found at DOI 10.6084/m9.figshare.28152620).

**Fig 4:**
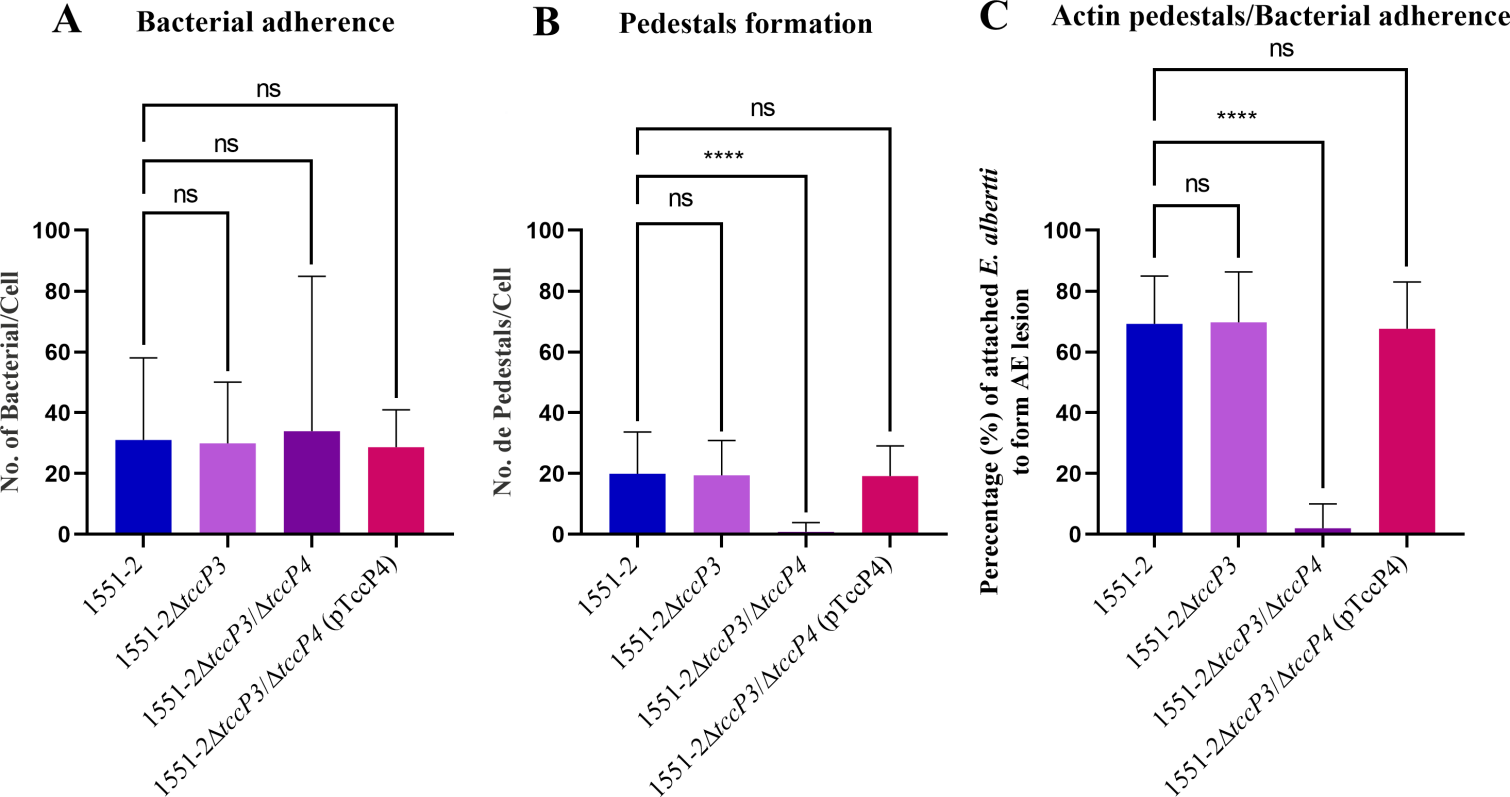
TccP4 is crucial for *E. albertii* 1551-2 to polymerize F-actin during AE lesion formation in infected Nck-null MEF cells. Fifty MEF cells, infected with wild-type, mutant, or complemented strains, were used to determine the number of adherent bacteria (**A**), the number of pedestals (**B**), and the percentage of adherent bacteria that formed pedestals (**C**). Note that the mutation in the *tccP4* gene did not affect the ability of the double mutant 1551-2Δ*tccP3*/Δ*tccP4* to adhere to the host cells. However, it directly affected its ability to trigger F-actin recruitment, which is necessary for the formation of pedestals characteristic of the AE lesion. ns = non-significant, ****P < 0.0001.

**Fig 5:**
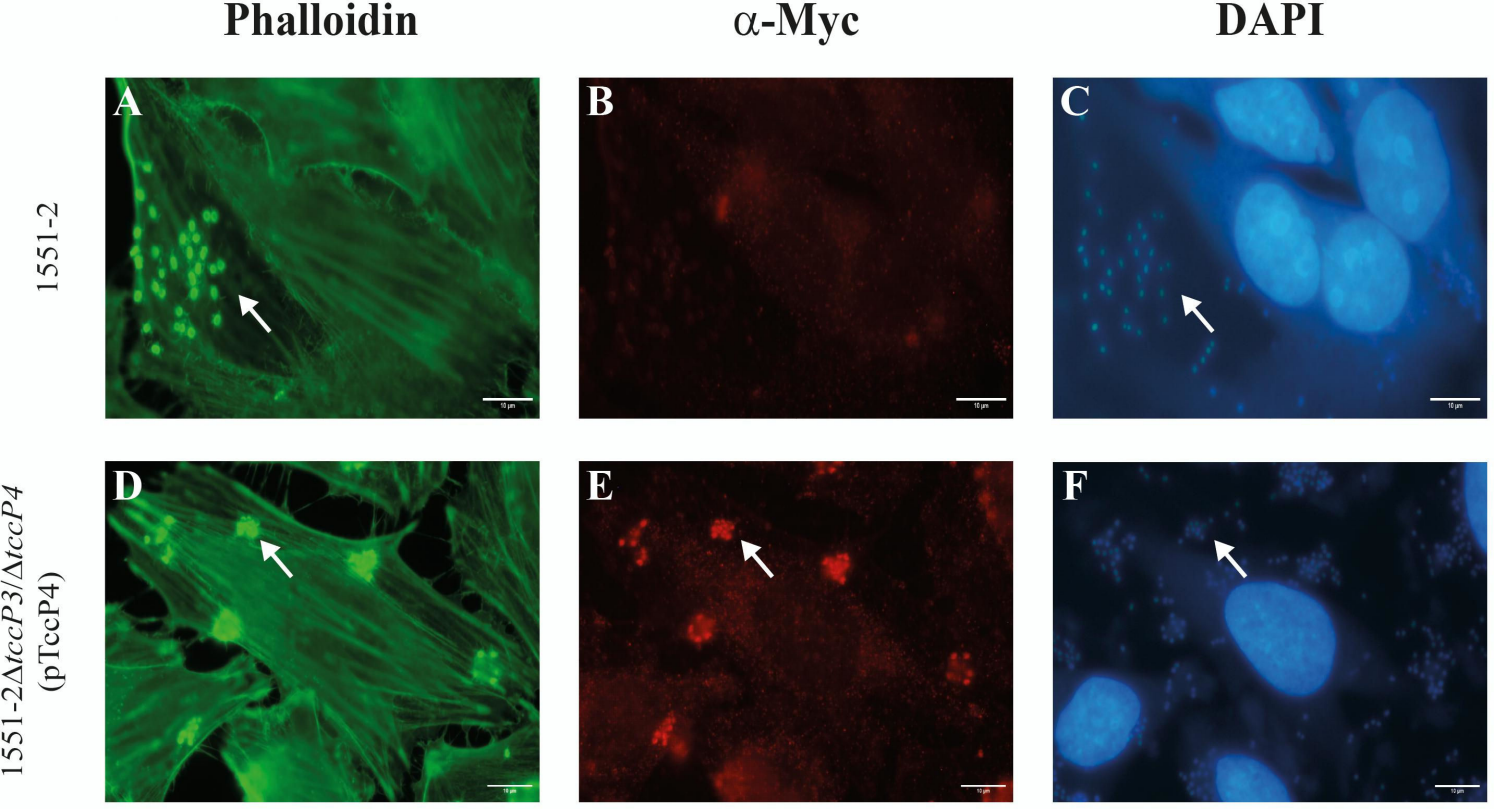
Colocalization of the recombinant TccP4-Myc protein with polymerized F-actin underneath adhered bacteria in infected HeLa cells. The co-localization of TccP4-Myc recombinant protein with polymerized F-actin, in infected epithelial HeLa cells, is indicated by arrows. Note that the TccP4-Myc recombinant protein was produced only by 1551-2Δ*tccP3*/Δ*tccP4*(pTccP4) complemented strain (**E**). Scale bar: 10 µm.

To better represent the results described above, quantitative assays of bacterial adherence and formation of F-actin-rich pedestals were carried out in Nck-null MEF cells. First, we confirmed that the ability of the 1551-2Δ*tccP3* mutant to induce F-actin polymerization in infected Nck-null MEF cells did not differ from that of the wild-type. By contrast, the lack of TccP4 led to a reduction of approximately 96% in the ability of the 1551-2Δ*tccP3/tccP4* double mutant strain to induce F-actin-rich pedestals formation in the infected Nck-null MEF cells when compared with the wild-type 1551-2 *E. albertii* strain (*P* < 0.0001) (Fig. 4). The ability to form F-actin-rich pedestals was quantitatively restored in the complemented 1551-2Δ*tccP3/tccP4*(pTccP4) strain (Fig. 4B and C). Of note, the mutation in the *tccP4* gene did not affect the adherence ability of the 1551-2Δ*tccP3/tccP4* double mutant strain (Fig. 4A). Moreover, we showed that the recombinant TccP4-Myc protein, produced by the 1551-2Δ*tccP3/tccP4*(pTccP4) complemented strain, colocalized with the polymerized F-actin, detected underneath the adherent bacteria, in infected HeLa cells (Fig. 5).

### TccP4 acts in F-actin recruitment in Nck-null cells infected with the EHEC O157:H7 strain EDL933

To test the hypothesis that TccP4 could replace TccP1 in F-actin recruitment for AE lesion formation in MEF Nck-null cells infected with the EHEC O157:H7 EDL933 strain, we constructed a *tccP*/*espF_U_* mutant strain (termed EDL933Δ*tccP*) and further transformed the mutant strain with the pKC471 or pTccP4 plasmids, the former of which carry the *tccP1* gene. The wild-type EDL933 as well as the derived strains exhibited similar growth rates, as evidenced by the bacterial growth curves (Fig. S2B; supplemental material is found at DOI 10.6084/m9.figshare.28152620).

As expected, we observed that the deletion of the *tccP1* gene abolished the ability of the EDL933Δ*tccP1* mutant strain to induce AE lesion formation in infected MEF Nck-null cells. Moreover, this phenotype was restored when the EDL933Δ*tccP1* mutant was transformed with pKC471 as well as with pTccP4 (Fig. S5; supplemental material is found at DOI 10.6084/m9.figshare.28152620).

### The *tccP4* gene is the most frequent *tccP*-encoding gene subtype present in *E. albertii* strains

An *in silico* screening for the presence of genes encoding the distinct TccP subtypes (*tccP1*, *tccP2*, *tccP3,* and *tccP4*) in a collection of 637 sequenced *E. albertii* strains revealed that the *tccP4* was the most frequent subtype detected (71.0%), followed by *tccP1* (61.9%), *tccP2* (15.2%), and *tccP3* (3.5%) (Table 2), while in 65 *E. albertii* strains (10.2%) no TccP-encoding gene was detected (Table S2; supplemental material is found at DOI 10.6084/m9.figshare.28152620). In addition, 330 *E. albertii* strains (51.8%) harbored two or three *tccP* subtypes concomitantly (Table S2; supplemental material is found at DOI 10.6084/m9.figshare.28152620), with the most common combination being *tccP1 +tccP4* (35.9%). Curiously, *tccP4* was not found in any of the genomes of other species of the *Enterobacteriaceae* family investigated, including *E. coli* (data not shown).

**TABLE 2:** Phylogenetic distribution of *tccP* genes in 637 genome-sequenced *E. albertii* strains

BAPS cluster^*a*^	No. of strains	Distinct TccP-encoding genes subtypes detected:			
		*tccP1*	*tccP2*	*tccP3*	*tccP4*
**1**	103	19 (18.4%)	31 (30.1%)	22 (21.4%)	71 (68.9%)
**2**	25	0 (0.0%)	15 (60.0%)	0 (0.0%)	25 (100.0%)
**3**	56	56 (100.0%)	31 (55.4%)	0 (0.0%)	55 (98.2%)
**4**	58	21 (36.2%)	0 (0.0%)	0 (0.0%)	5 (8.6%)
**5**	30	24 (80.0%)	0 (0.0%)	0 (0.0%)	3 (10.0%)
**6**	37	0 (0.0%)	1 (2.7%)	0 (0.0%)	32 (86.5%)
**7**	29	0 (0.0%)	0 (0.0%)	0 (0.0%)	27 (93.1%)
**8**	164	152 (92.7%)	19 (11.6%)	0 (0.0%)	144 (87.8%)
**9**	135	122 (90.4%)	0 (0.0%)	0 (0.0%)	90 (66.7%)
**Total**	637	394 (61.9%)	97 (15.2%)	22 (3.5%)	452 (71.0%)

^*a*^ BAPS: Bayesian Analysis of Population Structure.

**Fig 6:**
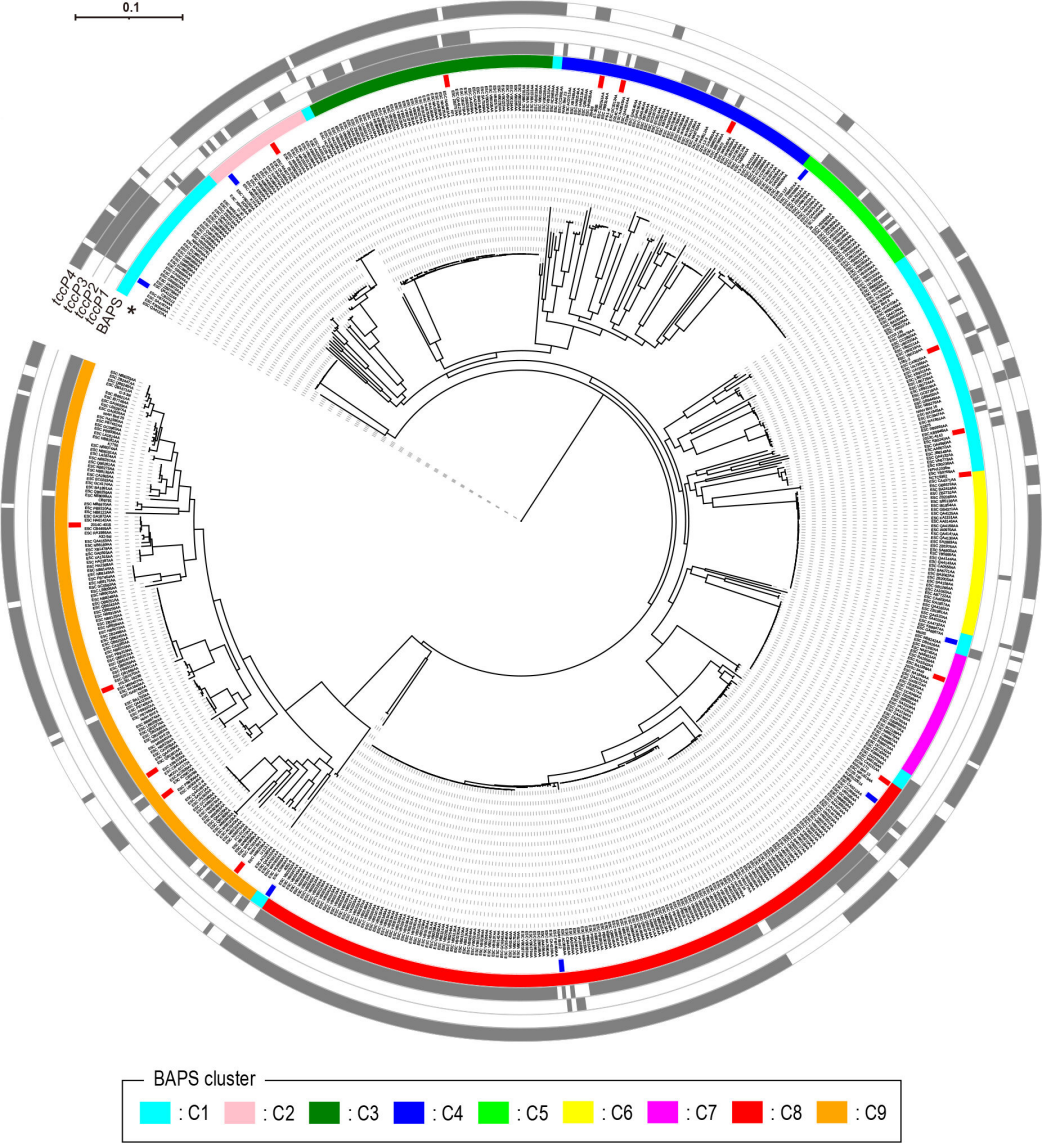
Phylogenetic relationships of the 637 genome-sequenced *E. albertii* strains analyzed in this study and the distribution of *tccP* genes in these strains. A maximum likelihood tree was constructed based on the 93,457 SNPs identified in the core genes ( = 1,879) of the 637 *E. albertii* genomes analyzed. The tree was rooted by *E. coli* K-12 strain MG1655 and strain names are indicated at each tip. BAPS clusters and the presence/absence of *tccP1*, *tccP2*, *tccP3*, and *tccP4* genes are shown in the outer circles. An asterisk indicates the innermost circle, where strains used for genetic structure comparison of *tccP4*-containing chromosome regions (shown in Fig. 7) are highlighted. In the innermost circle, strains for which only draft genome sequences were available are indicated in blue, while strains with the complete genome are indicated in red. Of importance, *tccP4* was the most frequent *tccP* subtype detected and found in all BAPS clusters.

The phylogenetic analysis of the 637 *E. albertii* genomes used in this study confirmed the existence of two major clades, referred to as clade 1 and clade 2, in the *E. albertii* population. Furthermore, Bayesian Analysis of Population Structure (BAPS) subdivided the *E. albertii* strains analyzed in 9 BAPS clusters (C1–C9), with 8 of them belonging to clade 1 (C1–C8) and one (C9) to clade 2 (Fig. 6; Fig. S6; supplemental material is found at DOI 10.6084/m9.figshare.28152620). The distribution of *tccP* genes in the *E. albertii* genomes analyzed revealed that *tccP1*, *tccP2*, and *tccP3* were distributed in limited BAPS clusters, while *tccP4* was found in all BAPS clusters identified (Table 2; Fig. 6). Notably, four *E. albertii* strains (ESC_QA4137AA, ESC_KB0840AA, ESC_SA4787AA, and ESC_FB7444AA) that were separated early from other strains harbored only the *tccP4* gene (Fig. 6; Fig. S6; supplemental material is found at DOI 10.6084/m9.figshare.28152620). This result, together with the wide distribution of the *tccP4* gene, suggests that *E. albertii* acquired *tccP4* at the very early stage of *E. albertii* diversification.

### The *tccP4*-containing chromosome region possesses a highly variable genetic organization

**Fig 7:**
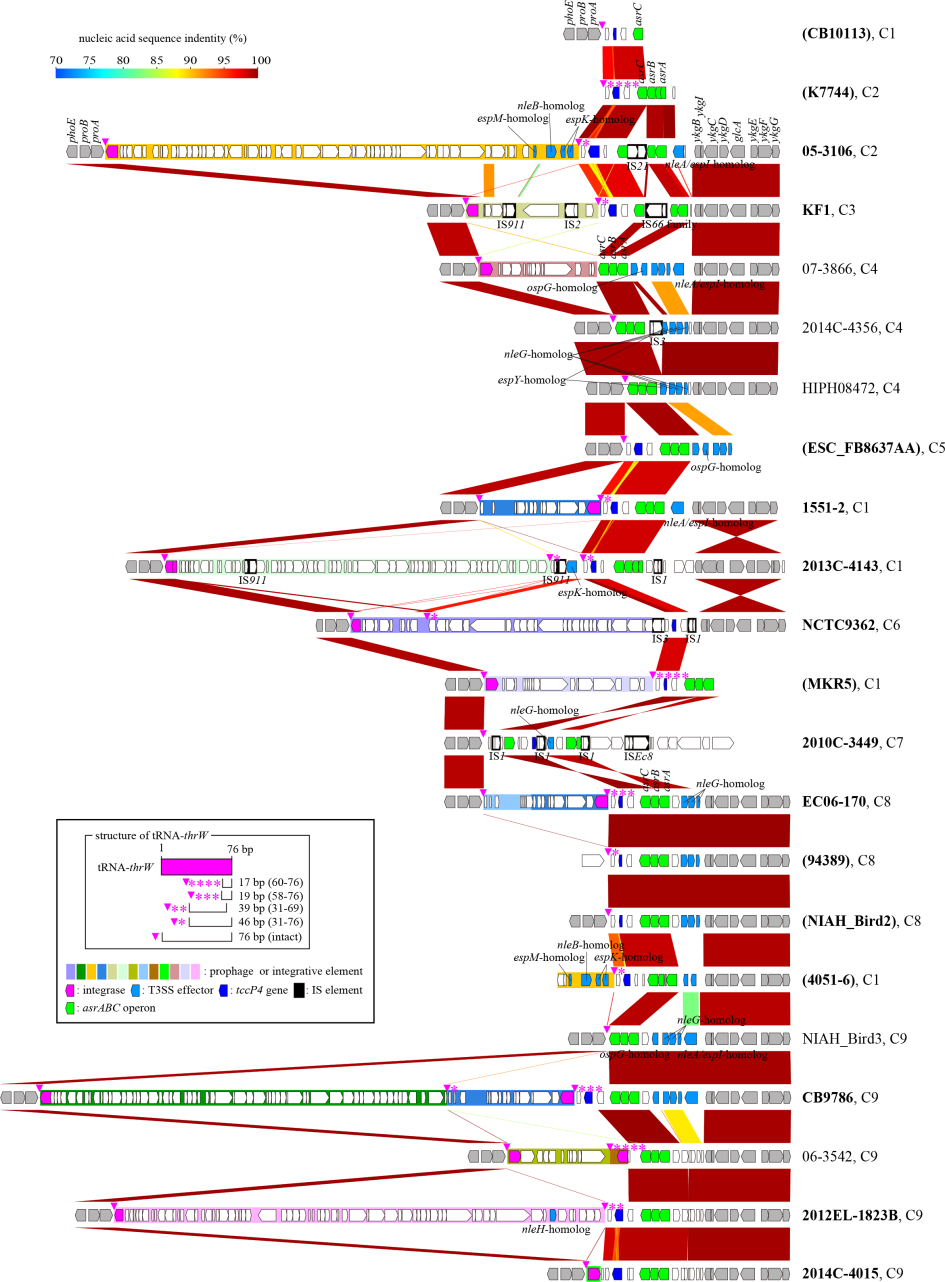
Genetic structure comparison of the *tccP4*-containing chromosome regions of 16 *tccP4*-positive *E. albertii* strains and the analogous regions of five *tccP4*-negative strains. BAPS clusters of each strain (**C1–C9**) are indicated after the strain name. *tccP4*-positive strains are indicated in bold, and strains, for which only draft genome sequences were available, are indicated by parenthesis. Note that in all *E. albertii* strains analyzed *tccP4* was present on the chromosome and close to the tRNA-*thrW* gene located between the *proAB* and *asrABC* operons. The genetic organization of this region was highly variable among the *E. albertii* strains evaluated.

To gain better insights into how *E. albertii* strains acquired *tccP4*, we performed the structural comparison of the *tccP4*-containing genomic regions of 16 *tccP4*-positive and the analogous genomic regions of five *tccP4*-negative strains, which were selected from different BAPS clusters. In this analysis, we observed that in all *E. albertii* strains evaluated the *tccP4* was present on the chromosome and close to the tRNA-*thrW* gene, located between the *proAB* and *asrABC* operons (Fig. 7). Note that the lengths of *tccP4* genes remarkably differed among the *E. albertii* strains due to the difference in the numbers of PRRs, ranging from one to six repeats. Moreover, we observed that the genetic organization of this region was highly variable among the *E. albertii* strains analyzed.

A variety of prophages and integrative elements, or traces of their integration, were observed integrated into the tRNA-*thrW* gene, even in some *tccP4*-negative strains. Of importance, the *tccP4* gene does not appear to be located on these mobile genetic elements (Fig. 7; Fig. S7; supplemental material is found at DOI 10.6084/m9.figshare.28152620). In addition, the genetic structure of this region has been more complicated in some *E. albertii* strains due to the insertion of several types of insertion sequences (ISs) and local genomic inversions. In the face of these findings, it was not possible to predict the ancestral genetic structure of this region and identify the genetic event involved in the acquisition of the *tccP4* gene.

## DISCUSSION

The AE lesion formation on the surface of epithelial cells infected by *E. albertii* strains is the main virulence strategy used by this pathogen to cause diarrheal disease in the human host (5, 7). The polymerization of F-actin, underneath adherent bacteria, is a key step in the AE lesion formation and involves the recruitment and activation of several eukaryotic components. Tir protein from EHEC of serotype O157:H7 lacks the equivalent of EPEC TirY_474_, and, consequently, does not recruit the eukaryotic protein Nck to polymerize F-actin for the AE lesion formation, a fact that intrigued researchers trying to understand the bacterial and eukaryotic proteins required for AE lesion formation induced by EHEC O157:H7. This question began to be answered when two independent research groups identified a bacterial protein, translocated to the eukaryotic cells by T3SS and termed TccP/EspF_U_ (referred to as TccP1 in this study), which is necessary for F-actin polymerization during the AE lesion formation by EHEC O157:H7 in a scenario independent of Nck (17, 20). After the discovery of TccP/EspF_U_ (TccP1), two additional subtypes of this adaptor protein— TccP2 and TccP3—were identified in the typical EPEC strain B171 and the *E. albertii* strain 1551-2, respectively (23, 24). The observation that the *E. albertii* 1551-2 strain can recruit F-actin in a scenario lacking Nck, and independently of TccP3, motivated us to further explore the potential for novel TccP subtypes in the 1551-2 strain, which culminated in the identification of TccP4.

The proline-rich effector TccP/EspF_U_ activates the eukaryotic actin nucleation-promoting factor N-WASP but does not bind Tir directly (31, 32). Further studies identified that the IRTKS eukaryotic protein binds to the TccP/EspF_U_ effector via its SH3 domain, while the IRSp53/MIM homology domain (IMD) of the IRTKS binds to the motif NPY_458_ of the C-terminal portion of Tir, thus bridging Tir and the adaptor effector TccP/EspF_U_ (21). TccP/EspF_U_ can bind to eukaryotic proteins, such as IRTKS and N-WASP, through specific motifs present in PRRs, leading to the recruitment of F-actin and subsequent AE lesion formation on the surface of infected epithelial cells (15). Progressive deletions within TccP/EspF_U_ revealed that the presence of the N-terminal translocation signal and at least two PRRs are the minimum requirements necessary for triggering F-actin polymerization in EHEC-infected epithelial cells (32). Drawing a parallel with what was described above, the observation that TccP4 harbored an N-terminal type 3 secretion system (T3SS) signal sequence and three PRRs led us to hypothesize that this effector could be functional after being translocated to the infected epithelial cells, a fact that was evidenced through the experimental approaches employed in this study.

The first 20 amino acids of the PRR domain comprise the portion of the EHEC O157:H7 TccP/EspF_U_ effector that binds to the autoinhibitory GTPase binding domain (GBD) in the N-WASP proteins, leading to its activation (33). Comparing the first 20 amino acids of the PRR1 of TccP4 and the PRR5 of the EHEC O157:H7 TccP/EspF_U_, we observed that they differ by only two amino acids. Taking the PRR5 of TccP/EspF_U_ as a reference, we observed the substitution of methionine (M) at position 9 by leucine (L), both with hydrophobic side chains; and the substitution of alanine (A), an amino acid with a hydrophobic side chain, at position 20 by glutamic acid (E), an amino acid with a negatively charged side chain. Considering that TccP4 was crucial for F-actin polymerization in Nck-null MEF cells infected by *E. albertii* 1551-2, these amino acid substitutions do not affect TccP4 functionality.

If, on the one hand, the N-terminal portion of TccP/EspF_U_ activates N-WASP (32, 33), the C-terminal portion binds to the IRTKS (26), acting as a link between these two eukaryotic proteins in the F-actin recruitment necessary for the AE lesion formation in infected epithelial cells. The interaction of TccP/EspF_U_ with the SH3 domain of the IRTKS/IRSp53 involves two adjacent PxxP motifs present in the C-terminal portion of the TccP/EspF_U_ adaptor protein, thus explaining the high affinity and selectivity of this interaction (26). These two PxxP motifs are conserved in the C-terminal portion of the three PRRs of the TccP4 protein, thus suggesting that, like TccP/EspF_U_ of EHEC O157:H7, TccP4 has a high affinity to bind to IRTKS/IRSp53 in the F-actin recruitment cascade. This comparative scenario with TccP4 (from the 1551-2 strain) and TccP/EspF_U_ (from EHEC O157:H7) indicates that both adaptor proteins act similarly in the F-actin recruitment process for the AE lesion formation.

The collective analysis of the data from the present study and previous publications from our laboratories (24, 25) allowed us to conclude that the *E. albertii* 1551-2 strain can polymerize F-actin using the Nck-dependent, as well as the Nck-independent pathways. The simultaneous use of both pathways for F-actin polymerization was first identified in a typical EPEC strain of serotype O119:H6 (34) and later observed in several typical and atypical EPEC strains of distinct serotypes (35–37). However, to date, studies with *in vivo* experimental models have not demonstrated that the coexistence of two distinct pathways for F-actin polymerization, during AE lesion formation, would result in bacteria that are more pathogenic for the host (38). However, we can hypothesize that the coexistence of two distinct pathways involved in the establishment of the AE lesion may represent a way to persevere this virulence characteristic in cases where the bacteria undergo mutations and/or genetic rearrangements that culminate in the modification and/or loss of genes responsible for encoding proteins involved in the establishment of this phenotype by one of the pathways.

Our analysis of the phylogenetic relationship of 637 genome-sequenced *E. albertii* strains and the distribution of the *tccP* genes in these strains provided interesting findings on the process of *tccP4* acquisition by *E. albertii*. Although several *tccP* subtypes were present in many *E. albertii* strains, the *tccP4* gene was the most frequent and observed in all *E. albertii* lineages identified (BAPS clusters C1-C9). Interestingly, the *tccP4* gene was detected in *E. albertii* strains that were first separated from other *E. albertii*. These findings added to the evidence that *tccP4* is located at the same chromosome locus in the *E. albertii* strains analyzed, suggesting that this gene was acquired at a very early stage during the diversification of *E. albertii* from its ancestor. In this regard, it is intriguing to note that the *tccP4* gene, or its close homolog, has not been found in *E. coli* strains, as well as in other bacteria of the *Enterobacteriaceae* family sequenced so far.

The highly variable genetic organization of the *tccP4*-containing regions and the presence of several mobile genetic elements in this region were also important findings, because although the genetic event involved in the *tccP4* acquisition by *E. albertii* was not predicted due to this variation, repeated genomic deletion mediated by these genetic elements may explain the lack of *tccP4* in *E. albertii* strains.

In conclusion, we identified a novel TccP subtype—termed in the present study as TccP4—and demonstrated that this protein is required for the recruitment of F-actin during the AE lesion formation in infected cells by the *E. albertii* 1551-2 strain. Furthermore, the gene encoding this TccP subtype unique to *E. albertii* is widely distributed in this species and suggests that the *tccP4* gene was acquired at a very early stage during the diversification process of *E. albertii*.

## MATERIALS AND METHODS

### Bacterial strains, plasmids, and genomes used in this study

The *E. albertii* 1551-2 strain was isolated from a diarrheic child at 23 months of age, during an epidemiological study performed at the Federal University of São Paulo (UNIFESP)—Brazil, in 1989 (27, 28, 39). All bacterial strains and plasmids used in the present study are listed in Table 1. The strains were routinely grown in Lysogeny Broth (LB) at 37°C and kept in LB supplemented with 30% glycerol at −80°C.

For *in silico* analysis, a total of 637 genome sequences of *E. albertii* strains registered in the EnteroBase website v1.2.0 (https://enterobase.warwick.ac.uk) (40) were used, after excluding 38 strains from the 675 strains registered as *E. albertii* (accessed on the 1st of May 2024) due to the lack of genome sequence information, low completeness (<95%) or high contamination (>5%) as estimated by CheckM (41). The final set of *E. albertii* strains used for *in silico* analysis is shown in Table S3 (Supplemental material is found at DOI 10.6084/m9.figshare.28152620).

### Phylogenetic analysis and amino acid sequence comparison of TccP4 with known TccP subtypes

The genome sequence of strain 1551-2 (accession number CP025317.1) was searched by tblastn using the amino acid sequences of TccP family proteins (TccP1, TccP2, and TccP3) listed in Table S4 as queries (Supplemental material is found at DOI 10.6084/m9.figshare.28152620). Coding sequences of TccP subtypes were manually annotated with GENETYX-MAC Version 16.0.9 (GENETYX Corp., Japan). The criterion of screening and subtyping was >97% amino acid sequence identity to the N-terminal 56 amino-acids sequence of any of the known TccP family proteins.

A maximum likelihood tree was built using the JTT matrix-based model (42) and analyzed using the MEGA11 software (43) to compare the amino acid sequence of the TccP4 effector from the 1551-2 strain (accession number: AUS64398.1) with those of other TccP subtypes previously described (Table S5; supplemental material is found at DOI 10.6084/m9.figshare.28152620). Subsequently, six representative amino acid sequences of the distinct TccP subtypes were used to be aligned using the ClustalW tool in MEGA 11 software. Percentage sequence identities between TccP subtypes were determined using blastp (https://blast.ncbi.nlm.nih.gov/Blast.cgi?PAGE=Proteins). The predicted molecular weight of the TccP4 protein was calculated using Expasy (https://web.expasy.org/cgi-bin/compute_pi/pi_tool).

### Deletion mutant construction and *in trans* complementation

A *tccP4* deletion mutant strain was constructed by the one-step allelic exchange recombination method (44), using the thermosensitive plasmid pKOBEG-Apra^R^, which carries lambda Red recombination genes (30). First, the 1551-2Δ*tccP3* strain (24) was transformed by electroporation with the pKOBEG-Apra^R^, and strains carrying this plasmid were selected on LB agar containing 100 µg/mL of apramycin after incubation for approximately 18 hours at 30°C.

Then, a DNA fragment harboring the gene encoding zeocin resistance (*ble*) flanked by 50 base pairs (bp) of the 5′ and 3′ ends of *tccP4* was prepared by polymerase chain reaction (PCR), using the primers tccP4-zeo-F and tccP4-zeo-R and the genomic DNA of the 1711-4Δ*fliC* mutant strain (29) as a template for the *ble* gene amplification. All primer sequences and conditions used in the PCR for DNA amplification are described in Table 3. Next, this DNA fragment was electroporated into strain 1551-2Δ*tccP3*, containing pKOBEG, and the recombinant bacteria were selected on LB low salt agar plates supplemented with 60 µg/mL of zeocin. To obtain *tccP4*-deletion mutants, bacteria recovered from zeocin-containing LB plates were screened for the loss of *tccP4* and the presence of *ble* using the set of primers tccP4-F/tccP4-R and zeo-F/zeo-R, respectively (Table 3).

**TABLE 3:** Primers used for PCR amplification

Primer identification	Primer sequence (5′’→ 3′)	PCR conditions:		Fragment size (bp)	Reference
		Annealing temperature	Extension time		
tccP4-zeo-F	ATGATTAAGGGCATTATTTCTTTTTTTTCTGGTATTCCTCGCGGCATAACGTCATCGCTTGCATTAGAAAGG	55°C	45 sec	659	This study
tccP4-zeo-R	TTACAGCTTCATATCTGGATGAAGTGCACCATTTAACACCGGCGGTGGCGGAATGATGCAGAGATGTAAG				
tccP4-F	ACCACGACATAAAGCCACCG	67°C	45 sec	540	This study
tccP4-R	GGAGCAGGGGGTATACATTCAG				
zeo-F	GTCATCGCTTGCATTAGAAAGG	55°C	45 sec	559	(45)
zeo-R	GAATGATGCAGAGATGTAAG				
tccP4-Flank-F	AAAAAAACCTCCACCGCC	55°C	1 min	965	This study
tccP4-Flank-R	AAGCCAGGTTAAACATATCAGG				
tccP4-XbaI-F	CTCTCTTCTAGAAATGAATATCGCAACATACCAA	55°C	45 sec	781	This study
tccP4-BamHI-R	CGCGCGGATCCCAGCTTCATATCTGGATGAAGT				
tccP/EHEC-zeo-F	GCTGATTACATATCTATCGGAGAACTAAAATTACTTATAAGGTAATCTGCGTCATCGCTTGCATTAGAAAGG	55°C	45 sec	659	This study
tccP/EHEC-zeo-R	AATAACCGGTAACTGTCAGGTCAGAGCTAATATAGGTAATTATATTATAAGAATGATGCAGAGATGTAAG				

Primers flanking the *tccP4* gene were then designed (tccP4-flank-F and tccP4-flank-R) (Table 3) and used to amplify a DNA fragment that was subsequently sequenced at the Biotechnology Institute/UNESP/Botucatu (https://www.ibtec.unesp.br/#!/servicos68/sequenciamento-sanger/) to confirm the correct insertion of the *ble* gene replacing *tccP4*. Once the loss of *tccP4*, pKOBEG was eliminated by growing the double mutant strain (1551-2Δ*tccP3/tccP4*) at 42°C in the presence of 60 µg/mL of zeocin.

To complement the double mutant 1551-2Δ*tccP3/tccP4* with the *tccP4* gene, a DNA fragment, from 100 bp upstream of the ATG start codon until just before the *tccP4* ORF stop codon, was amplified with primers tccP4-XbaI-F and tccP4-BamHI-R (Table 3) and cloned into XbaI and BamHI sites of the pKC471 plasmid (Table 1), thus creating the recombinant plasmid termed pTccP4. This recombinant plasmid (pTccP4) was electroporated into the double mutant to generate the complemented 1551-2Δ*tccP3/tccP4* (pTccP4) strain (Table 1).

Growth curves were constructed with at least three independent experiments for 6 hours with the *E. albertii* 1551-2 strain and its respective mutants and the *tccP4* complemented strain. The strains were grown in 3 mL of LB broth for 18 h at 37°C, and subsequently transferred to 25 mL of LB at a 1:50 dilution, followed by incubation at 37°C under constant shaking (250 rpm). Aliquots were analyzed every 30 min for optical density (OD) reading under λ = 600 nm in an ELISA reader (BioTek, USA). Two-way ANOVA was used for statistical analysis, and *P* values ≤ 0.05 were considered statistically significant.

### Immunoblotting for detection of the TccP4-Myc recombinant protein

The bacterial strains were grown in 3 mL of LB medium at 37°C for 18 hours. The cultures were centrifuged at 3,500 × *g* for 15 minutes to harvest the bacteria. The supernatant was discarded, and the resulting pellet was resuspended in 200 µL of phosphate-buffered saline (PBS). The preparations were boiled at 95°C for 5 minutes, according to the methodology described by Laemmli (46). Then, 20 µL of the preparations were mixed with 20 µL of sample buffer for sodium dodecyl sulfate-polyacrylamide gel electrophoresis (SDS-PAGE) and boiled again to denature the proteins. Approximately 15 µL of each preparation was added to a 12% SDS-polyacrylamide gel and proteins were separated by electrophoresis at 110 V until the tracking dye in the sample buffer reached the end of the gel. Subsequently, the proteins were transferred to nitrocellulose membranes at 10 V for 45 min and blocked with 5% non-fat milk in PBS for approximately 18 hours (47). The membranes were probed first with 1:5,000-diluted mouse monoclonal anti-Myc antibody (clone 9E40 sc-40, Santa Cruz Biotechnology, USA), and then, with 1:4,000-diluted anti-mouse secondary antibody (Sigma-Aldrich, USA) conjugated with peroxidase, diluted 1:4,000. Both antibodies were diluted in PBS containing 5% non-fat milk and 0.05% Tween-20 (LGC Biotecnologia, Brazil). Finally, the protein bands were detected by West Pico PLUS Chemiluminescent Substrate (Thermo Fisher Scientific, USA) and visualized on the Amersham Imager 600 (GE Healthcare, USA) transilluminator.

### F-Actin pedestals quantification

HeLa and Nck-null MEF cells were routinely cultivated in Dulbecco’s modified Eagle medium (DMEM) (Sigma, USA) supplemented with 10% fetal bovine serum (FBS) (Gibco, USA) and 1% antibiotic mixture (penicillin—10,000 U/mL and streptomycin—10 mg/mL, ThermoFisher, USA) in an atmosphere of 5% CO_2_ at 37°C.

For the fluorescent actin staining (FAS) assay, 1 mL of a cell suspension, prepared with DMEM supplemented with 2% FBS and containing 1 × 10^5^ HeLa cells and 4 × 10^4^ Nck-null MEF cells (48), was added to each well of a 24-well microplate (TPP, Switzerland). Then, the wild-type *E. albertii* 1551-2 strain, and the mutant and complemented strains were grown overnight in LB at 37°C, and a volume of 20 µL of a bacterial culture containing approximately 10^7^ CFU (colony-forming units) was added to the cell preparations and incubated for a total period of 6 hours, with a washing step after 3 hours and replacement of the culture medium, in a 5% CO_2_ atmosphere. The coverslips were washed six times with PBS and fixed with 3% formaldehyde for 24 hours. After, the wells were washed twice with PBS, permeabilized with 1% Triton X-100 (INLAB, Brazil) in PBS, and incubated with a solution containing FITC-phalloidin (Life Technologies, USA) (diluted at 1:125) and DAPI 4′,6-diamidino-2-phenylindole dihydrochloride (Life Technologies, USA) (1:500), prepared in PBS. The coverslips were mounted and observed with the Olympus BX60 fluorescence microscope under an immersion oil objective (100×).

For quantification of the number of F-actin pedestals present in the Nck-null cells infected with the wild-type, mutants, and complemented strains, 50 Nck-null MEF cells infected with each of the tested strains were randomly selected, and the number of bacteria, as well as the number of F-actin pedestals, were counted. The statistical analyses were performed with GraphPad Prism Version 8.0.1. One-way ANOVA was used for the parametric data, followed by the post hoc Tukey HSD test. Kruskal-Wallis was used for non-parametric data followed by the post hoc Dunn. *P* values ≤ 0.05 were considered statistically significant.

### Immunofluorescence to demonstrate the colocalization of TccP4 and polymerized F-actin

Hela cells were infected with the strains 1551-2 and 1551-2Δ*tccP3*/Δ*tccP4* (pTccP4) and incubated for 6 hours in an atmosphere of 5% CO_2_. Then, the cells were washed three times with PBS, fixed with 3% formaldehyde, permeabilized with 1% Triton X-100 in PBS for 5 min, and blocked with PBS supplemented with 2% bovine serum albumin (BSA) for 1 hour.

For detection of the TccP4-Myc recombinant protein, the infected cells were probed with mouse monoclonal anti-Myc diluted 1:100 in PBS (clone 9E40 sc-40, Santa Cruz, Biotechnology, EUA) for 1 hour. After two washes with PBS, the preparations were probed with Alexa Fluor 568-goat anti-mouse secondary antibody (Molecular Probes) diluted 1:1,000 in PBS. Subsequently, polymerized F-actin and DNA were stained with FITC-phalloidin (1:125) (Life Technologies, Carlsbad, EUA) and DAPI (1:500) (Life Technologies, Carlsbad, EUA) in PBS, respectively. The preparations were visualized with an Olympus BX60 fluorescence microscope under oil immersion.

### Mutant construction and *in trans* complementation in EDL933 strain

The EDL933Δ*tccP* mutant strain was constructed by the one-step allelic exchange recombination method (41), as described above, using the primers tccP/EHEC-zeo-F and tccP/EHEC-zeo-R (Table 3). The pKC471 and pTccP4 plasmids (Table 1) were introduced in the EDL933Δ*tccP* mutant strain by electroporation, generating the EDL933Δ*tccP*(pKC471) and EDL933Δ*tccP*(pTccP4) strains, respectively (Table 1). The growth rate of wild-type EDL933 and derivative strains were measured using growth curves performed as previously described.

### Identification and subtyping of *tccP* in *E. albertii*-sequenced genomes

To identify and subtype the TccP-encoding gene(s) present in the 637 genome-sequenced *E. albertii* strains, we performed tblastn using amino acid sequences of the representative TccP subtype as queries, as indicated in Table S4 (Supplemental material is found at DOI 10.6084/m9.figshare.28152620). The criterion for classifying TccP sequences as belonging to one of the four distinct subtypes was >97% amino acid sequence identity with the N-terminal 56 amino acid sequences of representative TccP family proteins used as queries.

### Search of *tccP4* in *E. coli* and other strains from the *Enterobacteriaceae* family

To determine whether TccP4 or its close homolog is encoded by *E. coli* and other strains from the *Enterobacteriaceae* family, tblastn analysis was performed on microbial genome blast (https://blast.ncbi.nlm.nih.gov/Blast.cgi?PROGRAM=tblastn&PAGE_TYPE=BlastSearch) using the amino acid sequence of TccP4 from 1551 to 2 strain as a query.

### Analysis of *tccP4*-containing regions

In 16 *tccP4*-positive and five *tccP4*-negative *E. albertii* genomes, the *tccP4*-containing regions in the *tccP4*-positive genomes and analogous regions in the *tccP4*-negative genomes were compared. Gene annotation was carried out with DFAST and manually curated using the *in silico* Molecular Cloning Genomics Edition software (IMC-GE ver. 8.61; *In Silico* Biology, Inc., Yokohama, Japan). Sequence comparison of the regions and dot-plot analysis were performed by blastn using the “CompareSequences” function of the GenomeMatcher software (49).

### Phylogenetic analysis of genome-sequenced *E. albertii* strains

Core gene SNP-based maximum likelihood (ML) tree of the 637 *E. albertii* strains was constructed as follows: the genome assemblies of a total of 637 strains (including strain 1551-2) were annotated using DFAST, and core genes (*n* = 1,879) were identified using Roary v3.13,0 (50) with a 90% amino acid sequence identity cut-off. Core gene SNPs (*n* = 93,457) were extracted using the core gene alignment tool in Roary and used as inputs for ML inference with RAxML v8 (51) and displayed and annotated using iTOL v4 (https://itol.embl.de) (52). Identical *E. albertii* genomes showing no SNPs were deduplicated (excluded strains were indicated in Table S3; supplemental material is found at DOI 10.6084/m9.figshare.28152620). BAPS clusters were identified using RhierBAPS (53).

## Data Availability

The raw data supporting the conclusions of this article will be made available by the authors, without undue reservation, to any qualified researcher.
